# Asxl1 C-terminal mutation perturbs neutrophil differentiation in zebrafish

**DOI:** 10.1038/s41375-021-01121-8

**Published:** 2021-01-22

**Authors:** Xiao Fang, Song’en Xu, Yiyue Zhang, Jin Xu, Zhibin Huang, Wei Liu, Shunqing Wang, Kuangyu Yen, Wenqing Zhang

**Affiliations:** 1 Division of Cell, Developmental and Integrative Biology, School of Medicine, South China University of Technology, Guangzhou, 510006 China; 2 Department of Hematology, Guangzhou First People’s Hospital, School of Medicine, South China University of Technology, Guangzhou, 510180 China; 3 Department of Developmental Biology, School of Basic Medical Sciences, Southern Medical University, Guangzhou, 510515 China; 4 State Key Laboratory of Experimental Hematology, National Clinical Research Center for Blood Diseases, Institute of Hematology & Blood Diseases Hospital, Chinese Academy of Medical Sciences & Peking Union Medical College, Tianjin, 300020 China

**Keywords:** Haematopoiesis, Haematological cancer

## Abstract

*ASXL1* is one of the most frequently mutated genes in malignant myeloid diseases. In patients with myeloid malignancies, *ASXL1* mutations are usually heterozygous frameshift or nonsense mutations leading to C-terminal truncation. Current disease models have predominantly total loss of ASXL1 or overexpressed C-terminal truncations. These models cannot fully recapitulate leukemogenesis and disease progression. We generated an endogenous C-terminal-truncated Asxl1 mutant in zebrafish that mimics human myeloid malignancies. At the embryonic stage, neutrophil differentiation was explicitly blocked. At 6 months, mutants initially exhibited a myelodysplastic syndrome-like phenotype with neutrophilic dysplasia. At 1 year, about 13% of mutants further acquired the phenotype of monocytosis, which mimics chronic myelomonocytic leukemia, or increased progenitors, which mimics acute myeloid leukemia. These features are comparable to myeloid malignancy progression in humans. Furthermore, transcriptome analysis, inhibitor treatment, and rescue assays indicated that *asxl1*-induced neutrophilic dysplasia was associated with reduced expression of *bmi1a*, a subunit of polycomb repressive complex 1 and a reported myeloid leukemia-associated gene. Our model demonstrated that neutrophilic dysplasia caused by *asxl1* mutation is a foundation for the progression of myeloid malignancies, and illustrated a possible effect of the Asxl1-Bmi1a axis on regulating neutrophil development.

## Introduction

Myeloid malignancies are characterized by the proliferation and defective differentiation of myeloid progenitors [1, 2]. The main types of these malignancies are myelodysplastic syndrome (MDS), myeloproliferative neoplasms (MPNs), and acute myeloid leukemia (AML). Chronic myelomonocytic leukemia (CMML) exhibits features of both MPN and MDS [3]. MDS, MPNs, and CMML have an inherent risk of transforming to AML. Knowledge about the leukemogenic mechanisms of myeloid malignancies can be a basis for developing therapeutic options.

Patients with multiple myeloid malignancies [4] including MDS, CMML, and AML frequently harbor somatic mutations in addition of sex combs-like 1 (*ASXL1*). In patients, *ASXL1* mutations are usually heterozygous frameshift or nonsense mutations leading to C-terminal truncation [4] and loss of the ASXM2 domain and a PHD finger. Truncated *ASXL1* mutations independently predict poor outcomes and shorter overall survival for patients with CMML [5, 6].

To understand how *ASXL1* participates in normal hematopoiesis and leukemogenesis, numerous models of aberrant *ASXL1* expression have been described [7–11]. Mouse *Asxl1* knockout (KO) models exhibit multilineage dysplasia [7]. Whereas loss of *asxl1* in zebrafish is described as leading to the apoptosis of hematopoietic stem cells (HSCs), two 17-month adult *asxl1* mutants developed an AML-like phenotype [12]. Overexpression of C-terminally truncated ASXL1 in mice inhibits myeloid differentiation and induces MDS-like diseases [8, 10, 11]. To obtain a model more closely resembling human disease, heterozygous *Asxl1*^*G643fs*^ mutant knock-in mice were generated [13, 14]. These models have no obvious hematopoiesis disorders within 18 months, but show MDS/MPN-like disease by 18–24 months [13, 14]. Even with these models and their different phenotypes, the role of ASXL1 in hematopoiesis is still largely unknown.

Mechanistically, the ASXL1 protein is thought to mediate the balance between polycomb and trithorax functions by regulating histone modifications such as monoubiquitination of histone H2A at lysine 119 (H2AK119ub) [15, 16] and methylation of histone H3 at lysine 3 (H3K4me3) [10]. C-terminally truncated ASXL1 aberrantly enhances BAP1 function in deubiquitination of H2AK119ub [16–18], which is catalyzed by polycomb repressive complex 1 (PRC1) [19]. This hyperactive mutant ASXL1/BAP1 complex is thought to promote impairment of myeloid differentiation through inhibition of H2AK119ub at posterior *HOXA* genes and *IRF8*, encoding an essential transcription factor in myeloid lineages [18]. The ASXL1^G643fs^ mutation disrupts interactions with BMI1, a subunit of PRC1, and downregulated BMI1-driven H2AK119ub is observed in the derepressed *p16Ink4a*, leading in an MDS-like phenotype [14]. Hence, precisely how *ASXL1* regulates H2AK119ub remains unclear. Furthermore, no consistent evidence indicates which factor is more important in mutant ASXL1-induced leukemogenesis. We need a model that closely resembles disease development in patients to recapitulate potential leukemogenesis and disease progression with ASXL1 C-terminal mutations.

An increasing number of leukemia models have been developed in zebrafish (*Danio rerio*) [20]. With properties such as external fertilization and embryonic development, zebrafish are an excellent model to study hematopoiesis, especially in early embryogenesis. To elucidate the function of Asxl1 in hematopoiesis and the leukemogenic mechanisms of Asxl1-truncated mutants, we established an endogenous truncated mutant in zebrafish that is comparable to human disease. Our C-terminally truncated zebrafish *asxl1* mutant, unlike mouse models, has explicitly impaired neutrophil development. At 1 year, in addition to neutrophilic dysplasia, around 13% of the mutants had the phenotype of monocytosis, which resembles CMML, or increased myeloblasts, similar to AML. This leukemogenesis induced by the truncated mutant Asxl1 is transplantable. Furthermore, we found that expression of *bmi1a*, a subunit of PRC1, was decreased in *asxl1*^−/−^ fish and its reduction was associated with neutrophil dysplasia in our mutants. These results were demonstrated with transcriptome analysis, inhibitor treatment, and rescue assays. Our work showed that impaired neutrophil differentiation is fundamental for the progression of myeloid malignancies and illustrated the possible function of the Asxl1-Bmi1 axis in regulating neutrophil development.

## Materials and methods

All information on oligos and target sequences is in Supplementary Table 1.

### Zebrafish, embryo collection, and treatment

All experiments involving zebrafish were done in accordance with guidelines from the Institutional Animal Care and Use Committee of South China University of Technology. Wild-type AB strain zebrafish were raised and maintained under standard conditions [21]. Embryos were collected and staged according to Kimmel et al. [22].

### Generation of *asxl1*-mutant lines and validation

CRISPR/Cas9 was used to create *asxl1* mutants. Cas9 mRNA was synthesized using mMESSAGE mMACHINE mRNA transcription-synthesis kits (Ambion). Cas9 mRNA (0.1 ng/embryo) and guide RNA (0.05 ng/embryo) were injected into single-cell wild-type embryos. Mutant embryos were identified through genotyping (see Supplementary Methods) and allowed to develop to F0. We identified F0 fish with inheritable mutations. F1 progeny from F0 outcrosses was identified by genotyping and sequencing. Identified F1 and progeny were used for experiments. *asxl1*^+/+^ and *asxl1*^−/−^ embryos were generated and genotyped from heterozygous intercrosses.

### RNA extraction and qRT-PCR

RNA was extracted using TRIzol (Invitrogen) and reverse transcribed by M-MLV Reverse Transcriptase (Promega). qPCR was with FastStart SYBR Green Master (Roche) and fold-changes determined by ΔΔCt method. All reactions were normalized against *ef1α.* Melting-curve analysis confirmed the presence of single PCR products. Significance was determined using Student’s *t* tests with a significance threshold of *p* < 0.05.

### RNA-sequencing (RNA-seq) and bioinformatics

At 3 days postfertilization (dpf), *asxl1*^+/+^ and *asxl1*^−/−^ embryos from *asxl1*^+/−^ intercrosses were collected, and tails including caudal hematopoietic tissue were used for total RNA extraction. Each sample library was from around 20 tails. Sequencing libraries were prepared from poly-A selected RNA using NGS RNA Library Prep kits (Novogene). Sequencing was by an Illumina NovaSeq sequencing platform to obtain 2 × 150-bp pair-end reads. Sequencing quality was assessed using FastQC [23]. Reads were mapped onto the GRCz11 zebrafish reference genome using STAR (version 2.5.1b) with default setting [24]. Raw read counts for each Ensembl-annotated gene were calculated by Featurecounts [25]. EdgeR (version 3.26.5, false discovery rate (FDR) < 0.05; exact test) was used to call differentially expressed genes [26, 27]. Gene ontology (GO) for biological processes was enriched using the Metascape online tool [28].

### Inhibitor treatment

Embryos at 24 h postfertilization (hpf) were soaked in egg water containing UNC3866 (Selleckchem, S8359), PRT4165 (Selleckchem, S5315), CPI455 HCl (Selleckchem, S8287), or GSK J4 (Selleckchem, S7581).

## Results

### Zebrafish *asxl1* is expressed in hematopoietic cells, including myeloid cells

The *asxl1* and *asxl2* genes are zebrafish homologs of the *Drosophila* Asx gene. Mammalian and zebrafish Asxl proteins are evolutionarily conserved. Phylogenetic analysis clustered zebrafish Asxl1 with mouse and human ASXL1, but separately from groups of ASXL2 or ASXL3 protein sequences (Supplementary Fig. 1A). The amino acid sequences of zebrafish Asxl1 and Asxl2 share an overall 33 and 42% identity with human ASXL1 and ASXL2 orthologs. They share a conserved common domain architecture, including ASXN, ASXH, ASXM1, ASXM2, and PHD domains (Supplementary Fig. 1B). These results indicated that *asxl1/2* are authentic orthologs of mammalian *ASXL1/2*. The conserved structural domains suggest functional conservation of ASXL members among species during evolution.

Expression patterns of *asxl1* during embryonic development were detected by WISH (Supplementary Fig. 1C). In embryos, *asxl1* was expressed as early as the 1-cell stage, indicating the presence of maternal transcript. At 5.3 hpf, *asxl1* was ubiquitously expressed and at 18 hpf, *asxl1* was restricted to the developing brain, eye, anterior lateral mesoderm, and posterior lateral mesoderm. At 24 and 36 hpf, *asxl1* was specifically expressed in brain and eye. At 2 dpf, *asxl1* was mostly in the nervous system (Supplementary Fig. 1C).

To investigate if *asxl1* participates in hematopoiesis, we first examined if *asxl1* expresses in hematopoietic cells. We collected adult kidney marrow cells from wild type before further sorting myeloid and lymphoid cells by FACS. qRT-PCR confirmed *asxl1* expression in adult hematopoietic cells, with higher levels in myeloid cells (Supplementary Fig. 1D). Hence, *asxl1* was expressed in hematopoietic cells, including myeloid cells. The expression of *asxl1* in myeloid cells supported the hypothesis that *asxl1* participates in zebrafish hematopoiesis, including myelopoiesis.

### *asxl1* C-terminal truncation generates new zebrafish mutants

**Fig. 1:**
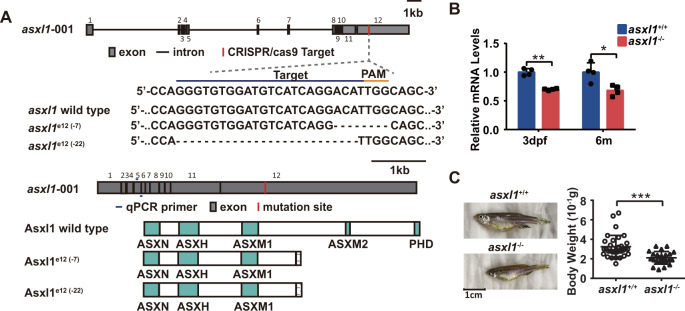
Genomic editing using CRISPR/Cas9 to generate *asxl1* C-terminally truncated mutant zebrafish. **A** Site-specific targeting for CRISPR/Cas9 cleavage within exon 12 of the zebrafish *asxl1* gene. Alignment of nucleotide sequences from wild-type and mutant *asxl1* alleles in *asxl1*^e12 (−7)^ and *asxl1*^e12 (−22)^ zebrafish lines. Dashes in DNA sequences are the nucleotides deleted during repair of CRISPR/Cas9-induced double-strand breaks. PAM protospacer adjacent motif. CRISPR/cas9-induced *asxl1* frameshift mutations predicted to lead to C-terminally truncated proteins. Truncated proteins predicted from mutant alleles *asxl1*^e12 (−7)^ and *asxl1*^e12 (−22)^ lack the last two domains (ASXM2 and PHD). **B** qRT-PCR comparing expression of *asxl1* in *asxl1*^+/+^ and mutant *asxl1*^−/−^ in 3 days postfertilization (dpf) larvae and adult kidney marrow (3 dpf, 3 dpf larvae tails, *n* ≥ 10 per group, performed with four replicates; 6 m, 6-month kidney marrow, *n* = 4 per genotype; two-tailed Student’s *t* test, *p < 0.05, **p < 0.01, ***p < 0.001; error bars, mean ± standard deviation (SD)). **C** Gross appearance of *asxl1*^−/−^ zebrafish compared with *asxl1*^+/+^ littermates (at 1.5 years). Body weights of *asxl1*^+/+^ and *asxl1*^−/^^−^ zebrafish (scale bar, 1 cm; *asxl1*^+/+^, *n* = 33; *asxl1*^−/−^, *n* = 24; two-tailed Student’s *t* test, *p < 0.05, **p < 0.01, ***p < 0.001, error bars, mean ± SD).

To examine if *asxl1* participates in hematopoiesis, we generated an *asxl1*-mutant line. CRISPR/Cas9 methods were used at exon 12 of the zebrafish *asxl1* gene to produce lines with premature stop codons, similar to mutations in myeloid malignancies (Fig. 1A). We obtained two independent *asxl1* alleles with exon 12 deletions of 7 bp in *asxl1*^e12 (−7)^ and 22 bp in *asxl1*^e12 (−22)^. Both deletions predicted to lead to stop codons (in amino acids 919 and 914, respectively) before the ASXM2 and PHD domains (Fig. 1A). These two mutants have the same phenotypes so unless specified, *asxl1*^e12 (−22)^ data are presented. qRT-PCR for *asxl1* expression in mutants showed decreased, which may be caused by nonsense-mediated decay (Fig. 1B). Most surviving *asxl1*^−/^^−^ fish displayed dwarfism and lower body weight (Fig. 1C). These results indicated that C-terminally truncated Asxl1 in zebrafish may lead to developmental abnormalities.

### Zebrafish *asxl1* mutants have impaired neutrophil differentiation as embryos

To investigate *asxl1* in zebrafish hematopoiesis, we detected expression of lineage markers in mutants from intercrosses of *asxl1* heterozygotes. First, the effects of *asxl1* C-terminal truncated mutation on primitive hematopoiesis (<24 hpf) were examined. Erythropoiesis (*gata1*) and myeloid progenitor (*pu.1*) markers were indistinguishable between *asxl1* mutants and siblings (Supplementary Fig. 2A). Additionally, a mature neutrophil marker (*lyz*) and intermediate neutrophilic progenitor marker for myeloperoxidase (*mpx*) were comparable between *asxl1* mutant and siblings (Supplementary Fig. 2A). Finally, we examined expression of a gene associated with macrophages (*mfap4*) (Supplementary Fig. 2A) and found it was not impaired in *asxl1* mutants. These data suggested that primitive hematopoiesis was not disrupted in *asxl1* mutants.

**Fig. 2:**
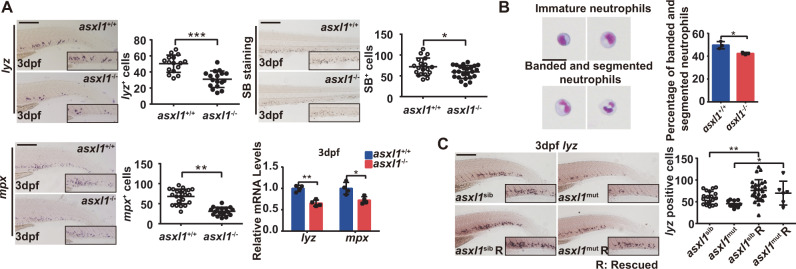
Zebrafish *asxl1* mutant has impaired neutrophil maturation. **A** Decreased *lyz* and *mpx* expression and decreased SB^+^ cells in *asxl1* mutants. WISH for *lyz*, *mpx*, and SB staining (3 dpf; scale bar, 200 μm; black boxes show enlarged details; *lyz* WISH, *asxl1*^+/+^, *n* = 16, *asxl1*^−/−^, *n* = 17; *mpx* WISH, *asxl1*^+/+^, *n* = 22, *asxl1*^−/−^, *n* = 17; SB staining, *asxl1*^+/+^, *n* = 19, *asxl1*^−/−^, *n* = 24; two-tailed Student’s *t* test, *p < 0.05, **p < 0.01, ***p < 0.001; error bars, mean ± SD). qRT-PCR comparison of *lyz* and *mpx* expression between wild type and mutant (3 dpf larvae, *n* ≥ 10 per group, performed with four replicates; two-tailed Student’s *t* test, *p < 0.05, **p < 0.01, ***p < 0.001; error bars, mean ± SD). **B** May–Grünwald–Giemsa staining of neutrophils from 4 dpf embryos and quantification of mature neutrophils by morphology. Neutrophils were collected from over 400 embryos of each genotype *asxl1*^+/+^ or *asxl1*^−/−^. After staining, 100 cells were randomly chosen for further calculation. This process was repeated three times for statistical tests (scale bars, 10 μm; two-tailed Student’s *t* test, *p < 0.05, **p < 0.01, ***p < 0.001; error bars, mean ± SD). **C** Reduced *lyz* expression rescued by *asxl1* expression. Left panel: WISH of *lyz* (3 dpf) after injection of *asxl1* mRNA. Right panel: quantification of *lyz*^+^ cells in *asxl1*^+/+^ and *asxl1*^−/−^ tails (0.1 ng mRNA/embryo; scale bar, 200 μm; black boxes show enlarged images; *asxl1*^sib^, *n* = 19, *asxl1*^mut^, *n* = 10, *asxl1*^sib^ R, *n* = 27, *asxl1*^mut^ = 6. R, mRNA injected; one-way ANOVA followed by LSD Fisher’s post hoc test, *p < 0.05, **p < 0.01, ***p < 0.001; error bars, mean ± SD).

We next characterized the biological function of *asxl1* in zebrafish definitive hematopoiesis by examining the effects of *asxl1* C-terminal truncated mutation on hematopoietic lineages. WISH and qRT-PCR at 3 dpf embryos (Fig. 2A) showed significantly reduced *lyz* and *mpx* transcripts. These observations suggested that *asxl1* may disrupt differentiation from myeloid progenitor to neutrophil. Supporting this, Sudan Black staining showed that SB^+^ granule containing mature neutrophils [29] were reduced in *asxl1* mutants (Fig. 2A). Furthermore, FACS-sorted neutrophils from *asxl1*^+/+^ and *asxl1*^−/^^−^ embryos were May–Grünwald–Giemsa stained to observe neutrophils morphology. Compared with wild type, the *asxl1* mutant exhibited a lower proportion of mature neutrophils, whose signature is banded and segmented nuclei (Fig. 2B). Expression of HSC marker (*c-myb*) (Supplementary Fig. 2B) and a lymphocyte marker (*rag1*) has no obvious change between *asxl1* mutants and siblings (Supplementary Fig. 2C). Expression of an erythrocyte (*hbbe1*) and a macrophage marker (*mfap4*) was not impaired in *asxl1* mutants (Supplementary Fig. 2C). Collectively, these results suggested that *asxl1* C-terminal truncation may specifically impair neutrophil differentiation. To determine if *asxl1* mutation caused the impaired neutrophil development, we performed rescue experiments. After injecting wild-type *asxl1* mRNA into 1-cell stage embryos, *lyz*^*+*^ cells increased in 3 dpf *asxl1* mutants and siblings (Fig. 2C). This result suggested a crucial role for *asxl1* in neutrophil differentiation.

### Six-month zebrafish *asxl1* mutants exhibit MDS-like phenotypes with neutrophilic dysplasia

**Fig. 3:**
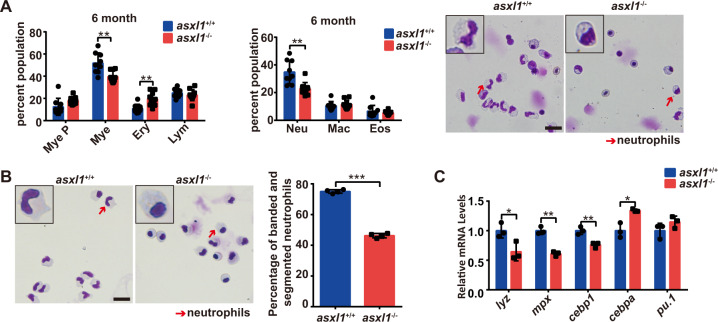
C-terminal *asxl1* mutation leads to MDS like with neutrophilic dysplasia in adult zebrafish. **A** Proportion of hematopoietic cells in whole kidney marrow of 6-month-old *asxl1*^+/+^, *asxl1*^−/−^ (*n* = 9 and *n* = 10, respectively; two-tailed Student’s *t* test, *p < 0.05, **p < 0.01, ***p < 0.001; error bars, mean ± SD). Mye P myeloid progenitors, Mye mature myeloid cells, Ery erythrocytes, Lym lymphocytes, Neu neutrophils, Mac macrophages, Eos eosinophils. May–Grünwald–Giemsa staining of whole kidney marrow from 6-month-old *asxl1*^+/+^ and *asxl1*^−/−^ (scale bar, 20 μm; black boxes show enlarged details of neutrophils; red arrows, neutrophils). **B** May–Grünwald–Giemsa staining of neutrophils from kidney marrow. Neutrophils were quantified by morphology. Neutrophils were collected from ten adult fish of each genotype *asxl1*^+/+^ or *asxl1*^−/−^. After staining, 500 cells were randomly chosen for further calculation. This process was repeated three times for statistical tests (scale bar, 20 μm; black boxes show enlarged details of neutrophils; red arrows, neutrophils; two-tailed Student’s *t* test, *p < 0.05, **p < 0.01, ***p < 0.001, error bars, mean ± SD). **C** qRT-PCR comparing expression of myeloid markers in sorted neutrophils from *asxl1*^+/+^ and *asxl1*^−/−^. Neutrophils were collected from ten adult fish of each genotype *asxl1*^+/+^ or *asxl1*^−/−^. qPCR was performed with three technical replicates (two-tailed Student’s *t* test, *p < 0.05, **p < 0.01, ***p < 0.001, error bars, mean ± SD) (color figure online).

The disruption of neutrophil development in *asxl1*^−/−^ larvae may lead to MDS-like phenotypes. To investigate this possibility, hematopoietic cells from kidney marrow, the functional ortholog of bone marrow, were examined in surviving homozygous mutants. Cytological staining of 6-month-old mutants indicated a significant decrease in the proportion of mature neutrophils accompanied by an increase in the proportion of erythroblasts (Fig. 3A). Neutrophils were FACS-sorted from *asxl1*^+/+^ and *asxl1*^−/−^ whole kidney marrow and May–Grünwald–Giemsa stained (Fig. 3B). Neutrophils from *asxl1*^−/−^ had lower percentages of banded and segmented mature neutrophils than wild type (Fig. 3B). Expression of neutrophil markers (*cebp1*, *mpx*, *lyz*) was lower in *asxl1* mutants than siblings, while expression of progenitor markers (*cebpa*) was higher (Fig. 3C). This finding indicated that *asxl1*-mutant neutrophils remained in an immature stage. The decrease and immaturity of neutrophils are typical of MDS with neutrophilic dysplasia [3].

### About 13% of 1-year-old zebrafish *asxl1* mutants progress to CMML- or AML-like disease

Both loss of *Asxl1* and transgenically expressed truncated *Asxl1* induce severe hematopoietic diseases in mice [7–11]. We therefore tested if adult *asxl1* zebrafish mutants developed severe myeloid malignancies. Kidney marrow from 55 1-year-old adult *asxl1*^−/−^ mutants was collected for blood cell count analysis (Supplementary Fig. 3A–F and Supplementary Table 2). These samples showed a significantly decreased proportion of neutrophils, similar to the 6-month mutants (Supplementary Fig. 3A). We observed a significantly increased proportion of monocytes/macrophages in *asxl1* mutants (Supplementary Fig. 3B) and a small increase in the proportion of myeloid progenitors (Supplementary Fig. 3C). Eosinophils and lymphocytes had no obvious change in *asxl1* mutants (Supplementary Fig. 3D, E). The significant increase in the proportion of erythroblasts in 6-month *asxl1* mutants was no longer observed in 1-year stage mutants (Supplementary Fig. 3F). This result may suggest the increased proportion of erythroblasts may be a temporary phenotype or found only in some samples. These data indicated that the *asxl1*-truncated mutation induced myeloid malignancies with neutrophilic dysplasia and monocytosis.

**Fig. 4:**
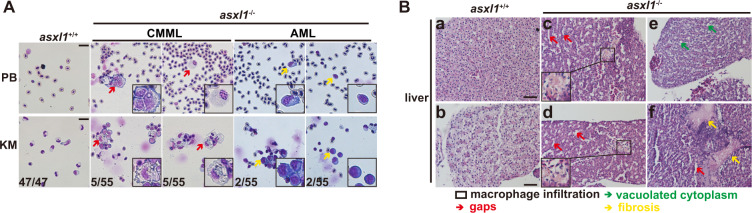
Zebrafish adult *asxl1* mutants progress to CMML-like or AML-like phenotypes. **A** May–Grünwald–Giemsa staining of peripheral blood (PB) and whole kidney marrow (KM) cells presentative five fish with CMML-like and two fish with AML-like phenotypes (scale bar, 20 μm; black boxes show enlarged details of macrophages and myeloid progenitors; red arrows, macrophages; yellow arrows, myeloid progenitors). **B** Hematoxylin and eosin staining of paraffin-embedded sections of liver from representative *asxl1*^+/+^ (a, b) and *asxl1*^−/−^ (c–f) (scale bar, 40 μm; black box, macrophage infiltration; green arrow, vacuolated cytoplasm; red arrow, gaps; yellow arrow, fibrosis) (color figure online).

Among the 55 1-year adult *asxl1* mutants, 5 had a severe monocytosis phenotype (defined as >20% of kidney marrow cells as monocytes/macrophages) with neutrophilic dysplasia and without excessive blasts (Supplementary Figs. 3A–C and 4A). These phenotypes are similar to CMML patients, who have increased numbers of monocytes with at least one lineage dysplasia, as classified in the World Health Organization (WHO) guidelines [3]. We also found two *asxl1* mutants with increased numbers of blasts. In these two mutants, the proportion of myeloid progenitors/blasts was over 40% of whole kidney marrow (Supplementary Fig. 3C and Fig. 4A), similar to features associated with human AML, as defined by WHO [30]. These data suggested that some of the *asxl1*-truncated zebrafish mutants further developed CMML-like or AML-like disease (Fig. 4A).

Histologic analysis of liver sections was performed on zebrafish with myeloid malignancies. CMML-like zebrafish showed perivascular macrophage infiltration (Fig. 4B—c, d). Liver parenchyma appeared abnormal in these mutants, with poorly demarcated cells (Fig. 4B). Cells with vacuolated cytoplasm were frequently observed in *asxl1*^−/−^ (Fig. 4B—e), similar to phenotypes as observed in by Gjini et al. [12]. Many gaps in the liver parenchyma were observed (Fig. 4B). Severe fibrosis was also observed in one of the CMML-like zebrafish (Fig. 4B—f). This phenomenon was similar to nonalcoholic fatty liver disease (NAFLD), where macrophages have a central role in progression [31]. The significantly increased proportion of monocytes/macrophages in our *asxl1* mutant may be a contributor to NAFLD-like progression in our *asxl1* mutants.

### C-terminally truncated Asxl1 mutation-induced leukemia cells are transplantable

**Fig. 5:**
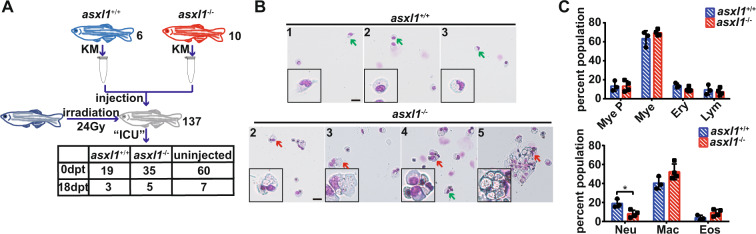
*asxl1* mutated cells intrinsically induce leukemogenesis and are transplantable. **A** Schematic of the experimental procedure. Wild-type fish were irradiated at 24 Gy 2 days before transplantation. Whole kidney marrow cells were prepared from 1-year stage *asxl1*^+/+^ and *asxl1*^−/−^ donors and were dilute to 10^5^/μL. Each recipient was injected with 3 μL donor kidney marrow cells. Transplanted fish were raised in sterile water. After 18 days, surviving fish were collected for analysis (dpt, days posttransplantation). **B** May–Grünwald–Giemsa staining of whole kidney marrow of successful transplanted fish (*asxl1*^+/+^, *n* = 3; *asxl1*^−/−^, *n* = 4; scale bar, 20 μm; black boxes show enlarged details of neutrophils and macrophages/monocytes; green arrows, neutrophils; red arrow, macrophages). **C** Proportion of hematopoietic cells in whole kidney marrow of transplanted fish (*asxl1*^+/+^, *n* = 3; *asxl1*^−/−^, *n* = 4; two-tailed Student’s *t* test, *p < 0.05, **p < 0.01, ***p < 0.001, error bars, mean ± SD). Mye P myeloid progenitors, Mye mature myeloid cells, Ery erythrocytes, Lym lymphocytes, Neu neutrophils, Mac macrophages, Eos eosinophils (color figure online).

To confirm if C-terminally truncated *asxl1* hematopoietic cells intrinsically induced leukemogenesis, we performed transplantation assays (Fig. 5A). *asxl1*^+/+^ and *asxl1*^−/−^ kidney marrow cells were intracardially injected into sublethally irradiated 9-month wild-type recipient fish. At 18 days posttransplantation, 3 *asxl1*^+/+^ and 5 *asxl1*^−/−^ recipients survived out of 19 and 35, respectively (Fig. 5A). The peripheral blood and kidney marrow cells from surviving recipients were collected for genotyping. Of five *asxl1*^−/−^ surviving recipients, four successfully repopulated (Supplementary Fig. 4A, B). Kidney marrow from these four successfully repopulated recipients was collected for further blood cell count analysis (Fig. 5B, C). The *asxl1*^−/−^ surviving recipients showed a significant decreased in the proportion of neutrophils (Fig. 5C). The neutrophilic dysplasia we observed in *asxl1*^−/−^ recipients was similar to the phenotype of 6-month and 1-year *asxl1* mutants (Fig. 3a and Supplementary Fig. 3A). A severe monocytosis phenotype (about 64% of kidney marrow cells were monocytes/macrophages) was observed in one *asxl1*^−/−^ recipient, and the morphology of these increased macrophages was aberrant (Fig. 5B—5). This phenotype is comparable to the CMML-like phenotype. These results demonstrated that our *asxl1* mutated cells intrinsically induced leukemogenesis and were transplantable.

### Transcriptome analysis shows disruption of neutrophil development

**Fig. 6:**
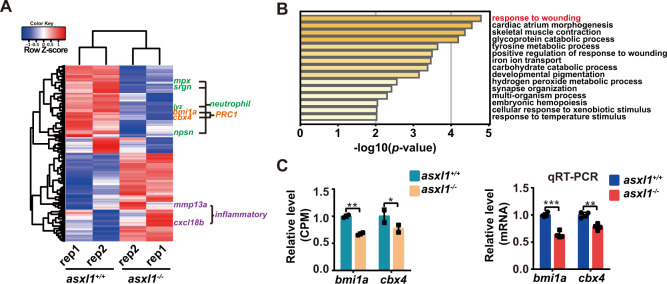
Expression of neutrophil and PRC1 markers decreased with *asxl1* mutation. **A** Heat map of differentially expressed genes (false discovery rate < 0.05; exact test) in 3 dpf *asxl1*^−/−^ compared to *asxl1*^+/+^ via RNA-seq. Neutrophil markers (green), PRC1 members (orange), and inflammatory response associated genes (purple) were highlighted. Rep replicate. **B** Gene ontology (GO) enrichment analysis of biological processes based on differentially expressed genes identified through RNA-seq (hypergeometric test). **C** Expression of *bmi1a* and *cbx4* from RNA-seq (*n* = 20 per replicate, CPM count-per-million; exact test, *false discovery rate (FDR) < 0.05, **FDR < 0.01, ***FDR < 0.001; error bars, mean ± SD). qRT-PCR comparing expression of *bmi1a* and *cbx4* in *asxl1*^+/+^ and *asxl1*^−/−^ in 3 dpf larvae (*n* ≥ 10 per group, performed with four replicates; two-tailed Student’s *t* test, *p < 0.05, **p < 0.01, ***p < 0.001; error bars, mean ± SD) (color figure online).

Our data showed that *asxl1* mutations induced dysregulation of myeloid differentiation, mainly displayed as similarities to myeloid malignancies with neutrophilic dysplasia. To understand the basis for impaired neutrophil differentiation in zebrafish *asxl1* mutants, we performed RNA-seq on 3 dpf *asxl1* mutants and its littermate controls. Our data showed 294 differentially expressed genes (FDR < 0.05, 146 downregulated genes, 148 upregulated genes, Supplementary Table 3, analysis detailed in “Methods”). Similar to the observed phenotypes, expression of all neutrophil markers (*lyz*, *mpx*, *npsn*, *srgn*) was downregulated (Fig. 6A and Supplementary Fig. 5A). Expression of myeloid progenitor (*pu.1* and *cebpa*) and macrophage (*mfap4* and *mpeg1*) markers was not impaired (Supplementary Fig. 5B). GO analysis showed that wounding response-related genes were significantly enriched in *asxl1* mutants, possibly because impaired neutrophils influenced the response to wounding (Fig. 6B). We also found that genes for an inflammatory cytokine (*cxcl18b*) and a matrix metalloproteinase (*mmp13a*) were upregulated in *asxl1* mutants (Fig. 6A), which may be from induction by the deficiency of neutrophil granzymes [32, 33]. Our transcriptome analysis mainly showed disruption of neutrophil development, also suggesting that neutrophil deficiency may stimulate expression of some inflammatory cytokines and enhance the inflammatory response.

### Expression of *bmi1a* and *cbx4* is disrupted in *asxl1* mutants

ASXL1 is thought to regulate H2AK119ub, which is ubiquitinated by PRC1 [34, 35]. ASXL1/2 mainly serve as cofactors for the H2AK119ub deubiquitinase BAP1 [16, 17, 36, 37], and overexpression of truncated ASXL1 reduces H2AK119ub [10]. From our RNA-seq data, the expression of *bmi1a* and *cbx4*, which encode members of PRC1, was downregulated in *asxl1* mutants (Fig. 6A, C). By qRT-PCR, we confirmed the reduced expression of *bmi1a* and *cbx4* in *asxl1* mutants (Fig. 6C). Our results indicated that the Asxl1 mutant may have disrupted expression of PRC1 complex components.

### Treatment with *bmi1a* and *cbx4* inhibitors impairs neutrophil development

**Fig. 7:**
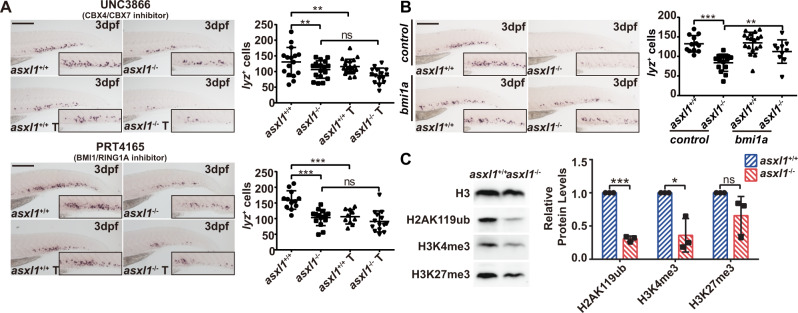
Inhibition of PRC1 components is associated with disruption of neutrophils development. **A** WISH for *lyz* (3 dpf) after UNC3866 treatment showing *lyz*^+^ cells decreased after 50 μM UNC3866 treatment. Quantification of *lyz*^+^ cells in *asxl1*^+/+^ and *asxl1*^−/−^ tails (scale bar, 200 μm; black boxes show enlarged images; *asxl1*^+/+^, *n* = 16, *asxl1*^−/−^, *n* = 18, *asxl1*^+/+^ T, *n* = 20, *asxl1*^−/−^ T, *n* = 15; one-way ANOVA followed by LSD Fisher’s post hoc test, *p < 0.05, **p < 0.01, ***p < 0.001; error bars, mean ± SD). T treated, ns not significant. WISH of *lyz* (3 dpf) after 10 μM PRT4165 treatment showing *lyz*^+^ cells decreased after PRT4165 treatment. Quantification of *lyz*^+^ cells in *asxl1*^+/+^ and *asxl1*^−/−^ tails (scale bar, 200 μm; black boxes show enlarged images; *asxl1*^+/+^, *n* = 12, *asxl1*^−/−^, *n* = 15, *asxl1*^+/+^ T, *n* = 10, *asxl1*^−/−^ T, *n* = 14; one-way ANOVA followed by LSD Fisher’s post hoc test, *p < 0.05, **p < 0.01, ***p < 0.001; error bars, mean ± SD). T treated, ns not significant. **B** WISH for *lyz* (3 dpf) after *bmi1a* mRNA injection showing reduced *lyz* expression was rescued by *bmi1a* mRNA injection. Quantification of *lyz*^+^ cells in *asxl1*^+/+^ and *asxl1*^−/−^ tails (about 0.1 ng mRNA/embryo; scale bar, 200 μm; black boxes show enlarged images; control: *asxl1*^+/+^, *n* = 12, *asxl1*^−/−^, *n* = 18; *bmi1a*: *asxl1*^+/+^, *n* = 18, *asxl1*^−/−^, *n* = 10; one-way ANOVA followed by LSD Fisher’s post hoc test, *p < 0.05, **p < 0.01, ***p < 0.001; error bars, mean ± SD; T treated, ns not significant). **C** Western blot of H2AK119ub, H3K4me3 and H3K27me3 in 2 dpf *asxl1*^+/+^ and *asxl1*^−/−^ embryos and quantification of Western blot data (input embryos: about ten embryos/well, performed with three replicates; two-tailed Student’s *t* test, ns not significant; error bars, mean ± SD, internal control, H3).

Studies showed that *bmi1a* is associated with the proliferative activity of granulocyte/macrophage progenitor (GMP) [38] and is a biomarker for hematologic malignancies [39]. This finding indicates that *bmi1a* may participate in myelopoiesis. We investigated if disruption of myelopoiesis in our *asxl1* mutant was associated with disruption of *bmi1a* and *cbx4* expression by testing if their inhibitors can phenocopy the *asxl1* mutation-caused neutrophilic dysplasia phenotype. In addition to H2AK119ub, H3K4me3 and H3K27me3 are also reported to be associated with ASXL1 [40–42]. Therefore, inhibitors targeting these demethylases were also employed. After testing for tolerance of the chemotherapeutics, the maximum doses were used: CPI455 (KDM5 H3K4me demethylase inhibitor, 40 μM), UNC3866 (CBX4/CBX7 inhibitor, 50 μM), PRT4165 (BMI1/RING1A inhibitor, 10 μM), and GSK J4 (JMJD3/UTX H3K27me demethylase inhibitor, 2 μM) (Supplementary Fig. 6A). After treatment of embryos with CBX4/CBX7 inhibitor or BMI1/RING1A inhibitor, *lyz*^+^ neutrophils decreased in *asxl1*^+/+^ compared with DMSO-treated controls (Fig. 7A). This result suggested that inhibition of Cbx4/Bmi1a phenocopied *asxl1*^−/−^. The KDM5 H3K4me demethylase inhibitor did not change the number of *lyz*^+^ cells in *asxl1*-mutant embryos (Supplementary Fig. 6B). The JMJD3/UTX H3K27me demethylase inhibitor increased the number of *lyz*^+^ cells in *asxl1*^+/+^ but not *asxl1*^−/−^ (Supplementary Fig. 6C). This result indicated that induction of *lyz*^+^ cells after H3K27me demethylase JMJD3/UTX inhibitor treatment in wild-type embryos may involve *asxl1*. These results are consistent with the hypothesis that a reduction in expression of PRC1 members *bmi1a* and *cbx4* may be associated with disruption of neutrophils in *asxl1*^−/−^ embryos.

### Impaired neutrophil development from *asxl1* mutations is rescued by *bmi1a* mRNA

To determine if impaired neutrophil differentiation was associated with decreased *bmi1a/cbx4* expression, we overexpressed *bmi1a* and *cbx4* in *asxl1* mutants by injecting *bmi1a/cbx4*-3xFLAG-P2A-DsRed mRNA (Supplementary Fig. 7A). Fluorescence indicated *bmi1a/cbx4* expression: DsRed was observed in embryos injected with *bmi1a/cbx4*-3xFLAG-P2A-DsRed mRNA compared to controls (Supplementary Fig. 7B). Expression of both *lyz* and SB^+^ neutrophils was significantly increased in *asxl1* mutants after injecting *bmi1a* mRNA (Fig. 7B and Supplementary Fig. 7C). Injection with *cbx4* mRNA led to almost complete embryonic lethality at 3 dpf and embryos exhibited an expanded heart field (Supplementary Fig. 7B). Our data showed that expression of *bmi1a* can rescue the *asxl1* mutation-induced phenotype and indicated that decreased *bmi1a* expression may be a mechanism by which neutrophils were disrupted in *asxl1*^−/−^ embryos.

BMI1 is reported to be required for the formation of PRC1 and for RING1A/1B ubiquitin ligase activity [43]. To check if the reduction in *bmi1a* also influenced H2AK119ub, we examined the global H2A ubiquitination state in *asxl1*-mutant zebrafish embryos. Western blots showed decreased global H2AK119ub (Fig. 7C). Global H3K4me3 and H3K27me3, which are reported to be associated with ASXL1 [8, 41, 42], were also detected. H3K4me3 was decreased in zebrafish *asxl1* mutants, whereas global H3K27me3 did not obviously change. Our data showed that an endogenous *asxl1*-truncated mutant mainly disrupted H2AK119ub and this disruption may be associated with reduced expression of genes encoding PRC1 members, *bmi1a* and *cbx4*.

## Discussion

### How might ASXL1 facilitate myeloid malignancies?

Patients with multiple myeloid malignancies frequently harbor an *ASXL1* mutation. Existing disease models mainly have total loss of ASXL1 [7, 9] or overexpression of a C-terminal truncation [8, 10, 11]. Loss of ASXL1 in mice is described to exhibit multilineage dysplasia [7, 9]. A zebrafish KO model is described to have reduced HSCs at 3 dpf [12]. In addition, C-terminally truncated ASXL1 can be detected in leukemia cells with an *ASXL1* mutation [42] and expression of ASXL1 with a C-terminal truncation may confer a gain-of-function by promoting BAP1 activity [18]. These results raised the possibility that a *ASXL1* mutation might confer a gain-of-function effect. Therefore, mouse models overexpressing C-terminally truncated ASXL1 were generated and showed dysregulation of multilineage differentiation [10, 11]. However, an overexpressing model may not fully recapitulate ASXL1 function because of protein overdose. Therefore, endogenous heterozygous *Asxl1*^G643fs^ mutant knock-in mice were generated and had no obvious hematopoiesis disorders within 18 months, but showed MDS/MPN-like disease by 18–24 months [13, 14].

In contrast to mouse models, zebrafish have external fertilization and embryonic development, allowing us to observe phenotypes starting from the embryonic stage. Our zebrafish with endogenous truncated *asxl1* mutants captured the most frequent C-terminal frameshift mutations in patients. This model had specifically impaired neutrophil differentiation without effects on other lineages including erythrocytes, macrophages, and lymphocytes. Furthermore, transcriptome analysis showed that zebrafish *asxl1* mutants exhibited predominantly dysregulation of neutrophil-specific genes. Our work complements a recent study employing single-cell RNA-seq that suggests the involvement of ASXL1 in neutrophil differentiation [44].

Our data showed that C-terminally truncated *asxl1* disrupted only neutrophil differentiation. The observed mild phenotype may be more comparable to clinical features since *ASXL1* mutations frequently coexist with other mutations [45]. This phenomenon complements previous studies showing that *Asxl1* mutation alone is not sufficient for leukemogenesis [13]. Moreover, we found that neutrophil disruption happened as early as the embryonic stage and persisted throughout the lifetime. At 6 months, mutants with Asxl1 truncations in our study mainly displayed MDS with neutrophilic dysplasia, which presented as refractory neutropenia. At 1 year, in addition to neutrophilic dysplasia, about 13% of mutants acquired other phenotypes of monocytosis that mimic CMML or increased myeloblasts similar to AML (Fig. 4). This result suggests a fundamental role for neutrophil disruption in myeloid malignancies progression.

However, further progression to CMML-like or AML-like phenotypes was observed only in some mutants, suggesting that *asxl1* mutations alone are sufficient for MDS-SLD but not for severe myeloid malignancies, with other events responsible for progression. Clonal evolution and the alteration of the hematopoietic niche might contribute to disease progression [1]. In addition, inflammation was recently reported to contribute to clonal hematopoiesis of indeterminate potential [46]. Chronic inflammation may lead to HSC exhaustion and aging, which may promote mutation [47–50]. Our transcriptome analysis showed a possible inflammatory response in our mutants. Mutant *asxl1*-induced neutrophilic dysplasia may induce persistent inflammation and increase the risk of myeloid malignancy progression. Further studies need to be done to investigate the mechanism of progression, and our model could provide an opportunity to investigate additional contributors to myeloid malignancy progression.

### How might *bmi1a* be an intermediate in neutrophil disruption?

Our data indicated that disruption of neutrophil development has a fundamental role in myeloid malignancy progression in *asxl1* mutants. We explored the potential mechanism. Previous studies show that an ASXL1^G643fs^ mutant interrupts its interaction with BMI1, leading to an MDS-like phenotype [14]. However, in our model, we observed reduced expression of *bmi1a*. Hence, ASXL1 mutants may not only block interaction with BMI1 but also disrupt its expression. Absence of *Bmi1* affects the proliferative activity of GMP, and *Bmi1*^−/−^ GMP has dispersed macrophage colonies [38]. This result indicates that *Bmi1* may participate in myeloid differentiation. Using a *bmi1a* inhibitor, we mimicked a neutrophil dysplasia phenotype (Fig. 7A). Supplementing with *bmi1a* mRNA rescued *asxl1*-induced neutrophil dysregulation (Fig. 7B). Hence, our work illustrated an alternative mechanism of Asxl1-Bmi1a axis on regulating neutrophil development.

The reduction in H2AK119ub is important in mutant ASXL1-induced leukemogenesis. Recent studies show that overexpression of C-terminally truncated ASXL1 (ASXL1-MT) and BAP1 leads to a hyperactive complex that subsequently upregulates *IRF8*, which is an essential transcription factor for myeloid lineage development [51–53], through H2AK119 deubiquitination. In addition, disrupted interaction between ASXL1^G643fs^ and BMI1 perturbs BMI1-driven H2AK119ub, further promoting an MDS-like phenotype. The reduced expression of PRC1 members, including BMI1 and CBX4, further contributes to the mutant *asxl1*-induced dysregulation of H2AK119ub. However, we are not suggesting that reduction of H2AK119ub blocks neutrophil differentiation. This H2AK119ub change could be a consequence of the reduced expression of *bmi1a* and *cbx4*. In any case, our data show a novel way for ASXL1 mutations to reduce H2AK119ub and raise the possibility that the Asxl1-Bmi1a axis is involved in neutrophil differentiation.

**Fig. 8:**
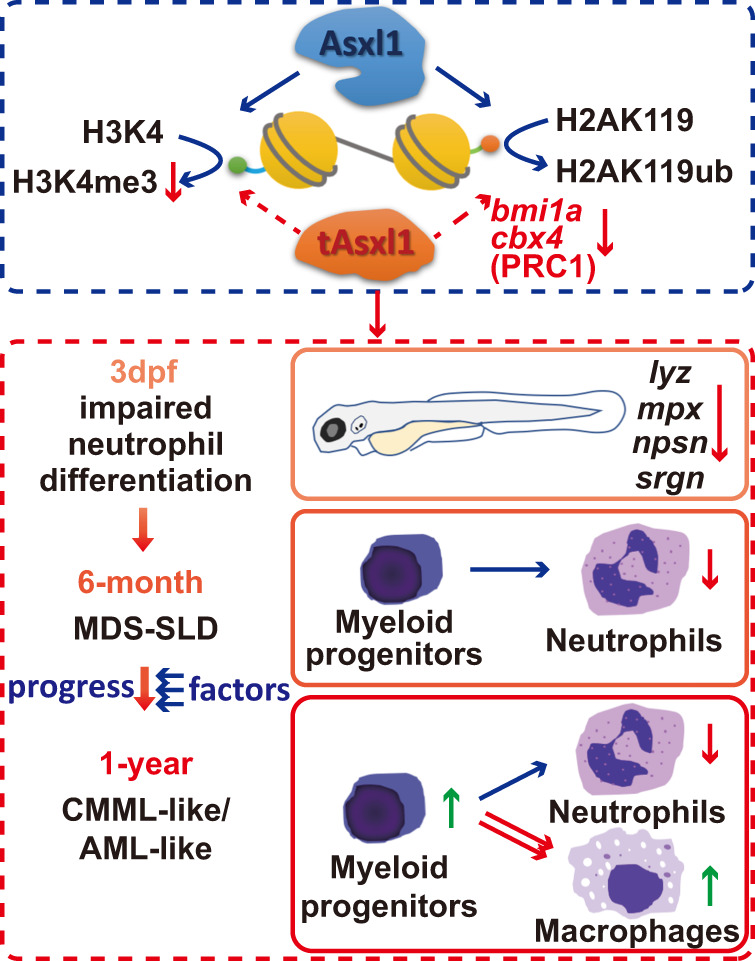
Schematic diagram of Asxl1 mutations resulting in myeloid malignancies in zebrafish. In our model, C-terminally truncated with *asxl1* mutations specifically blocked neutrophil differentiation. The mutants initially exhibited an MDS-like phenotype with neutrophilic dysplasia, and some mutants progressed to more severe phenotypes, similar to CMML/AML disease. Dysregulation of *bmi1a* may be a reason for leukemogenesis in *asxl1* mutants. Asxl1 wild-type Asxl1 protein, tAsxl1 truncated Asxl1 protein.

Taken together, results from this study show that endogenous truncated *asxl1* mutations result in impaired neutrophil differentiation. Our model revealed that neutrophilic dysplasia caused by *asxl1* mutations is a foundation for the progression of myeloid malignancies, and *bmi1a* dysregulation is associated with neutrophil dysplasia (Fig. 8). Zebrafish are an ideal model for large-scale chemical screening. Our endogenous truncated *asxl1* zebrafish model provides a new opportunity for a high-throughput drug-screening platform using whole organisms.

## Supplementary information

Supplemental materials

Supplemental table 1

Supplemental table 2

Supplemental table 3

## Data Availability

RNA-seq data are available at GEO under accession number GSE142214. For original data, please contact Xiao Fang: fangxiao@scut.edu.cn.
