# Supplementary material for: Asxl1 C-terminal mutation perturbs neutrophil differentiation in zebrafish
Source: Leukemia. 2021 Jan 22;35(8):2299–310. doi: 10.1038/s41375-021-01121-8 (PMC8324474; doi:10.1038/s41375-021-01121-8)

Supplemental Methods

Fluorescence-activated cell sorting (FACS) and May-Grünwald-Giemsa staining

Hematopoietic cells were obtained from whole kidney marrow from wild type AB, *asxl1*$^{+/+}$ and *asxl1*$^{-/-}$ adult zebrafish. Myeloid cells and lymphocytes were sorted based on physical characteristics of cell size and internal complexity (forward and side scatter). Purity of myeloid and lymphoid populations was confirmed by May-Grünwald-Giemsa staining[1] (purity of myeloid >90%; purity of lymphoid>80%).

To obtain neutrophils from *asxl1*$^{+/+}$ and *asxl1*$^{-/-}$, *asxl1* mutants were outcrossed with a Tg(*lyz*:DsRed)$^{+}$ line to specifically label the neutrophil population.[2] Tg(*lyz*:DsRed)$^{+}$; *asxl1*$^{+/+}$ and Tg(*lyz*:DsRed)$^{+}$; *asxl1*$^{-/-}$ embryos were collected and dissociated for fluorescence-activated cell sorting (FACS) as described previously.[3] Neutrophils of adult *asxl1*$^{+/+}$ and *asxl1*$^{-/-}$ were obtained from whole kidney marrow of Tg(*lyz*:DsRed)$^{+}$; *asxl1*$^{+/+}$ and Tg(*lyz*:DsRed)$^{+}$; *asxl1*$^{-/-}$. Neutrophils were identified by morphology by May-Grünwald-Giemsa staining.[1]

Cloning of *asxl1*, probe synthesis, and whole-mount in situ hybridization

asxl1 amplification products were cloned into pBluescript II SK (+) and used as templates to generate antisense or sense digoxigenin-labeled RNA probes. The RNA probes were prepared by *in vitro* transcription according to a standard protocol.[4] Whole-mount *in situ* hybridization (WISH) of zebrafish was performed at hybridization temperature 65°C[5] with sense probes as negative controls.

Cloning of *asxl1*, *bmi1a* and *cbx4* cDNA, in-vitro differentiation and rescue assay

asxl1 amplification products were cloned into pCS2+. *bmi1a* and *cbx4* cDNA amplification

products were cloned into modified pCS2+, which had inserts of P2A and a reporter gene. These vectors were used as templates to synthesize mRNA using mMESSAGE mMACHINE mRNA transcription-synthesis kits (Ambion). Synthetic mRNA was then microinjected into 1- to 2-cell stage embryos (*asxl1*$^{+/+}$ and *asxl1*$^{-/-}$). WISH was performed to detect the expression of *lyz* in 3 dpf embryos.

Genotyping

asxl1$^{+/+}$ and *asxl1*$^{-/-}$ embryos were generated from heterozygous intercrosses and identified by PCR and restriction enzyme digestion. The PCR primers were: *asxl1*_FP: 5'-TGAACCATCTCCCTCTTCTGTG-3'; *asxl1*_RP: 5'- GTCTGAGCCATGCTGGATAACT-3'. Restriction enzyme was Hpy188III (New England Biolabs), which digested PCR products amplified from genomic DNA of *asxl1*$^{+/+}$ but not *asxl1*$^{-/-}$.

Sudan black staining

Sudan black (SB) staining was as previously described.[6, 7]

Cytological analysis

Peripheral blood (PB) and kidney marrow were resuspended in ice-cold phosphate-buffered saline (PBS) with 5% fetal bovine serum. Resuspended cells were spun onto slides using cytospins at 400 rpm, 3 min. After May-Grünwald-Giemsa staining, blood cells of kidney marrow were calculated manually based on their morphologies[1]. Neutrophils, macrophages, eosinophils, myeloid progenitors, erythroblasts and lymphocytes were classified and counted. Over 500 cells were counted in each sample.

Western blots

Deyolked zebrafish embryos were homogenized with Cell Lysis Buffer (CST, #9803). Western

blotting was done according to standard procedures.[8] Antibodies were against H3 (Abcam, ab1791), H3K4me3 (Abcam, ab8580), H3K27me3 (Abcam, ab6002), or H2AK119ub (CST, #8240). Detection was with anti-rabbit or anti-mouse HRP-conjugated secondary antibodies (Abcam, ab6721 and ab6728, respectively) and High-sig ECL Western Blotting Substrate (Tanon).

Microscopy and imaging

Whole-mount images were captured with a Zeiss AXIO Zoom V16 with Axiocam 506 color camera. Blood cell counts and mounted embryos images were captured with a Zeiss Imager A2 with Axiocam 506 color camera.

Histology

1-year CMML-like and AML-like *asxl1*$^{-/-}$ and age-matched *asxl1*$^{+/+}$ were fixed in 4% paraformaldehyde at 4 °C at least 3 days. Fixed fish were decalcified by 20% EDTA (pH=8.0) about 7 days, then dehydrated by ethanol, cleared by xylene, and embedded in paraffin. Tissues were sectioned at 5 μm and stained with hematoxylin and eosin (H&E).

Transplantation assay

Whole kidney marrow cells were prepared from 1-year *asxl1*$^{+/+}$ and *asxl1*$^{-/-}$ fish. Two days after receiving a sublethal dose of 24 Gy, 9-month-old wild type recipients were intracardially injected with 0.3 million whole kidney marrow cells using a glass capillary needle (World Precision Instruments; 1B100-6). Transplanted fish were raised in sterile water.

Statistical analysis

Data were analyzed by SPSS software (version 22) using the Student's *t*-test for comparison between two groups and one-way ANOVA (with least significant difference adjustment) among multiple groups. Data are presented as mean ± standard deviation (SD). Significance was accepted

when $p < 0.05$. The specific statistical test for each experimental data is detailed in the associated figure legend.

Supplemental References

1. Carradice D, Lieschke GJ. Zebrafish in hematology: sushi or science? *Blood* 2008; **111**(7)**:** 3331-3342.

2. Hall C, Flores MV, Storm T, Crosier K, Crosier P. The zebrafish lysozyme C promoter drives myeloid-specific expression in transgenic fish. *BMC developmental biology* 2007 May 04; **7:** 42.

3. Manoli M, Driever W. Fluorescence-activated cell sorting (FACS) of fluorescently tagged cells from zebrafish larvae for RNA isolation. *Cold Spring Harbor protocols* 2012 Aug 1; **2012**(8).

4. Chitramuthu BP, Bennett HP. High resolution whole mount in situ hybridization within zebrafish embryos to study gene expression and function. *Journal of visualized experiments : JoVE* 2013 Oct 19; (80)**:** e50644.

5. Thisse C, Thisse B. High-resolution in situ hybridization to whole-mount zebrafish embryos. *Nature protocols* 2008; **3**(1)**:** 59-69.

6. Le Guyader D, Redd MJ, Colucci-Guyon E, Murayama E, Kissa K, Briolat V*, et al.* Origins and unconventional behavior of neutrophils in developing zebrafish. *Blood* 2008 2008-01-01 00:00:00; **111:** 132-141.

7. Lieschke GJ, Oates AC, Crowhurst MO, Ward AC, Layton JE. Morphologic and functional characterization of granulocytes and macrophages in embryonic and adult zebrafish. *Blood* 2001 2001-11-15 00:00:00; **98:** 3087-3096.

8. Westerfield M. *The Zebrafish Book: A Guide for the Laboratory Use of Zebrafish (Danio Rerio)*. University of Oregon Press, 2007.

Supplemental Figure Legends

Supplemental Figure 1. Expression of *asxl1* in zebrafish. (A) Phylogenetic tree of the full amino acid sequences of ASXL1 proteins in three species, built using the neighbor-joining method via MEGA 5.0. (B) Multiple alignments of *asxl1* conserved domains from human and zebrafish. Sequences in black indicate identical or similar residues. (C) Spatial-temporal expression pattern of *asxl1* in early-stage zebrafish embryos. Sense probe was the negative control. Representative figures from at least 3 separate experiments containing more than 10 embryos per experiment are shown. Lateral view, anterior to the left, dorsal upwards. Scale bar, 200 μm. (D) RT-qPCR of *asxl1* expression in FACS-sorted adult zebrafish blood cell populations (triplicate replicates of cDNA isolated from 10 wild type fish pooled kidney marrow samples, one-way ANOVA followed by LSD Fisher's post hoc test, *p<0.05, **p<0.01, ***p<0.001; error bars, mean ± SD.) WKM, whole kidney marrow; Mye, Myeloid cells; Lym, Lymphocytes.

Supplemental Figure 2. Primitive hematopoiesis and definitive erythrocytes, lymphocytes were not influenced in *asxl1* mutants. (A) WISH of hematopoiesis markers: myelopoiesis (*pu.1*, *lyz*, *mpx*, *mfap4*) and erythropoiesis (*gata1*) in primitive stage. (B) WISH of hematopoietic stem cell marker (*cmyb*). (C) WISH of hematopoiesis markers: macrophages (*mfap4*), erythrocytes (*hbbe1*) and lymphocytes (*rag1*) in definitive stage. Sample numbers are noted in the right corner of each panel. Scale bar, 200 μm.

Supplemental Figure 3. Myeloid disorders were observed by cytological staining. (A-F) Proportion of hematopoietic cells from whole kidney marrow samples of 1-year-old *asxl1*$^{+/+}$ fish (open circles) and *asxl1*$^{-/-}$ fish (gray filled circles), including with severe disease phenotypes (colored symbols); ns,

not significant (two-tailed Student's *t*-test, *p<0.05, **p<0.01, ***p<0.001; error bars, mean ± SD).

Supplemental Figure 4. *asxl1*$^{-/-}$ surviving recipients successfully repopulated. (A) Alignment of nucleotide sequences from *asxl1*$^{+/+}$ and *asxl1*$^{-/-}$ at mutation site. WT, wild type; MUT, mutant. WT reversed primer sequence is in blue; MUT forward primer sequence is in red. (B) Detection by PCR of wild type and mutated *asxl1* genomic DNA in recipients.

Supplemental Figure 5. Expression of neutrophil markers decreased with *asxl1* mutation. (A) Expression of neutrophil markers (*lyz, mpx, npsn* and *srgn*). (n=20 per replicate, CPM, count-per-million; exact test, *FDR < 0.05, ** FDR < 0.01, *** FDR < 0.001; error bars, mean ± SD). (B) Expression of myeloid progenitor markers (*pu.1 and cebpa*) and macrophage markers (*mfap4 and mpeg1*). (n=20 per replicate, CPM, count-per-million; exact test, *FDR < 0.05, ** FDR < 0.01, *** FDR < 0.001; error bars, mean ± SD).

Supplemental Figure 6. The effects of chemotherapy on zebrafish development. (A) The percentage of the embryos that developed to be normal or deformed depended on the chemotherapeutic concentration. Blue, CPI455; yellow, UNC3866, green, PRT4165; red, GSK J4. (n = 20 per concentration) (B) WISH of *lyz* (3dpf) after CPI-455 treatment showing *lyz*$^{+}$ cells had no obvious change after CPI-455 treatment. Quantification of *lyz*$^{+}$ cells in *asxl1*$^{+/+}$ and *asxl1*$^{-/-}$ tail (Scale bar, 200 μm; black boxes show enlarged images; *asxl1*$^{+/+}$, n = 39, *asxl1*$^{-/-}$, n = 29, *asxl1*$^{+/+}$ T, n = 31, *asxl1*$^{-/-}$ T, n = 21; one-way ANOVA followed by LSD Fisher's post hoc test, *p<0.05, **p<0.01, ***p<0.001; error bars, mean ± SD). (C) WISH of *lyz* (3 dpf) after GSK J4 treatment showing *lyz*$^{+}$

cells increased after GSK J4 treatment in *asxl1*$^{+/+}$ but not *asxl1*$^{-/-}$. Quantification of *lyz*$^{+}$ cells in *asxl1*$^{+/+}$ and *asxl1*$^{-/-}$ tail (scale bar, 200 μm; black boxes show enlarged images; *asxl1*$^{+/+}$, n = 17, *asxl1*$^{-/-}$, n = 18, *asxl1*$^{+/+}$ T, n = 10, *asxl1*$^{-/-}$ T, n = 13; one-way ANOVA followed by LSD Fisher's post hoc test, *p<0.05, **p<0.01, ***p<0.001; error bars, mean ± SD).

Supplemental Figure 7. SB^{+} neutrophils increased after *bmi1a* injection. (A) Schematic representation of *bmi1a*/*cbx4*-3xFLAG-P2A-DsRed mRNA. (B) Fluorescence indicated *bmi1a*/*cbx4* expression: DsRed was observed in embryos injected with *bmi1a*/*cbx4*-3xFLAG-P2A-DsRed mRNA compared to controls at 2 dpf. (White arrow indicates an expanded heart field after expression of *cbx4* mRNA; scale bar, 200 μm). (C) SB staining after *bmi1a* mRNA injection showing reduction in SB^{+} neutrophils was rescued by *bmi1a* mRNA injection. Quantification of SB^{+} neutrophils in *asxl1*$^{+/+}$ and *asxl1*$^{-/-}$ tail (about 0.1 ng mRNA/embryo; scale bar, 200 μm; control: *asxl1*$^{+/+}$, n = 15, *asxl1*$^{-/-}$, n = 9; +*bmi1a*: *asxl1*$^{+/+}$, n = 11, *asxl1*$^{-/-}$, n = 14; one-way ANOVA followed by LSD Fisher's post hoc test, *p<0.05, **p<0.01, ***p<0.001; error bars, mean ± SD).

Supplemental Fig 1. Expression of *asxl1* in zebrafish.
related to Fig 1

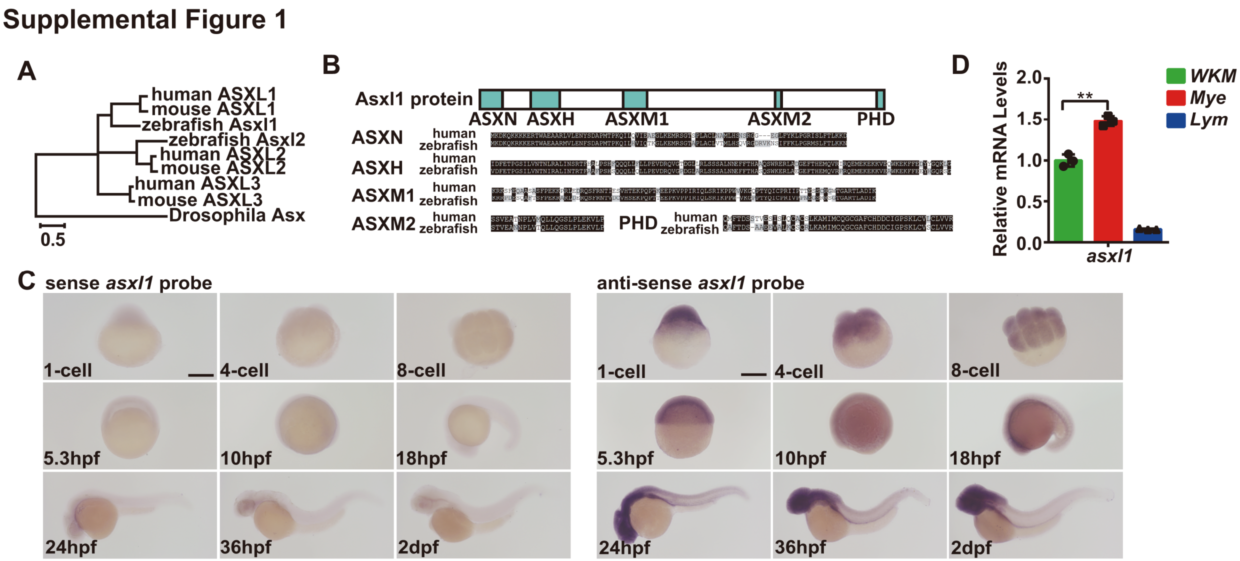

Supplemental Fig 2. Primitive hematopoiesis and definitive erythrocyte, lymphocytes were not influenced in *asxl1* mutant. related to Fig 2

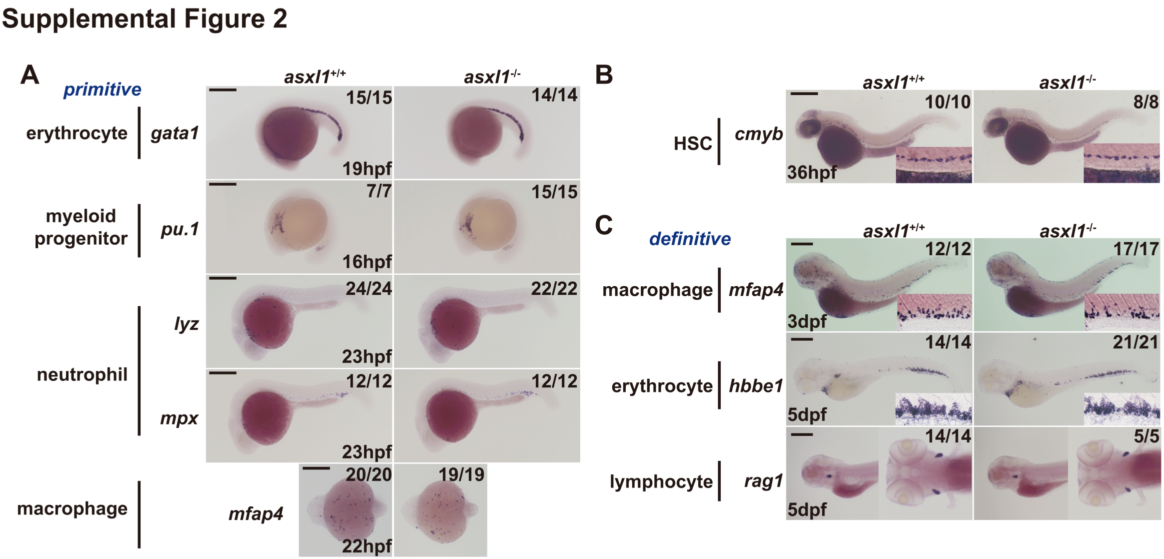

Supplemental Fig 3. Myeloid disorders were observed by cytological staining.
related to Fig 4

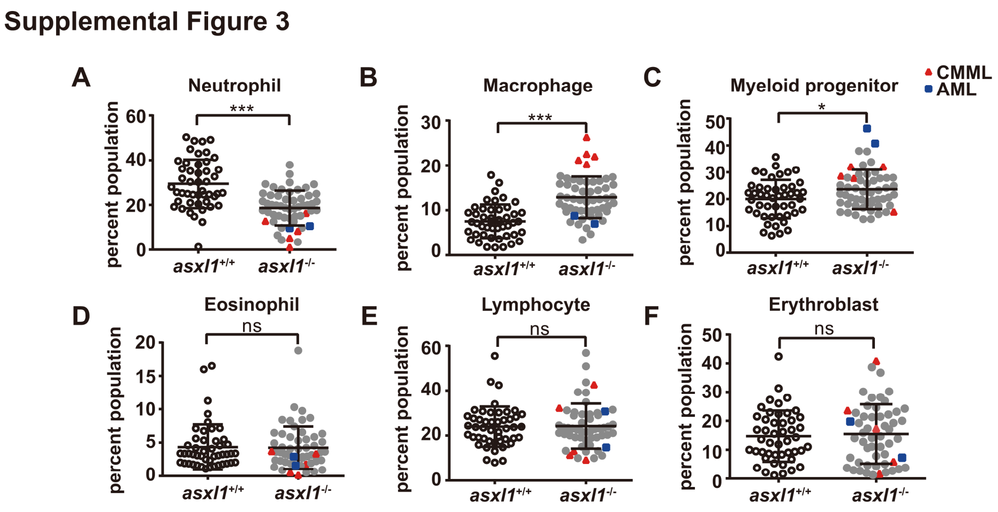

Supplemental Fig 4. *asxl1*$^{-/-}$ surviving recipients successfully repopulated. related to Fig 5

Supplemental Figure 4

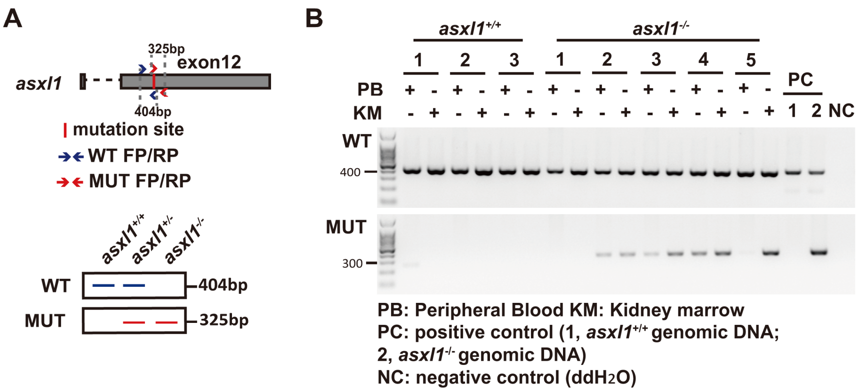

Supplemental Fig 5. Expression of neutrophil markers decreased with *asxl1* mutation. related to Fig 5

Supplemental Figure 5

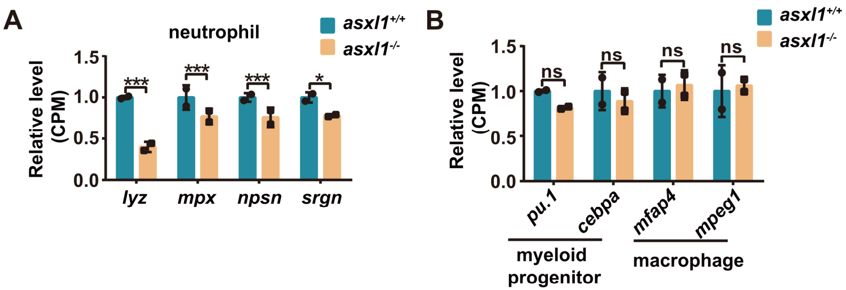

Supplemental Fig 6. The effects of chemotherapy on zebrafish development. related to Fig 6

Supplemental Figure 6

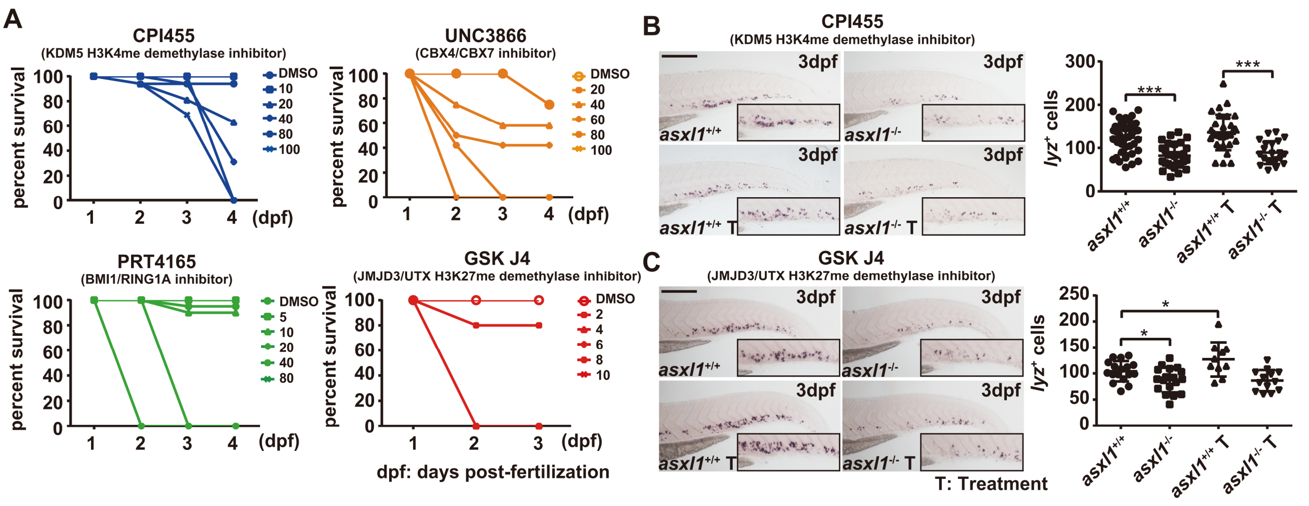

Supplemental Fig 7. SB+ neutrophils were increased after *bmi1a* injection. related to Fig 7

Supplemental Figure 7

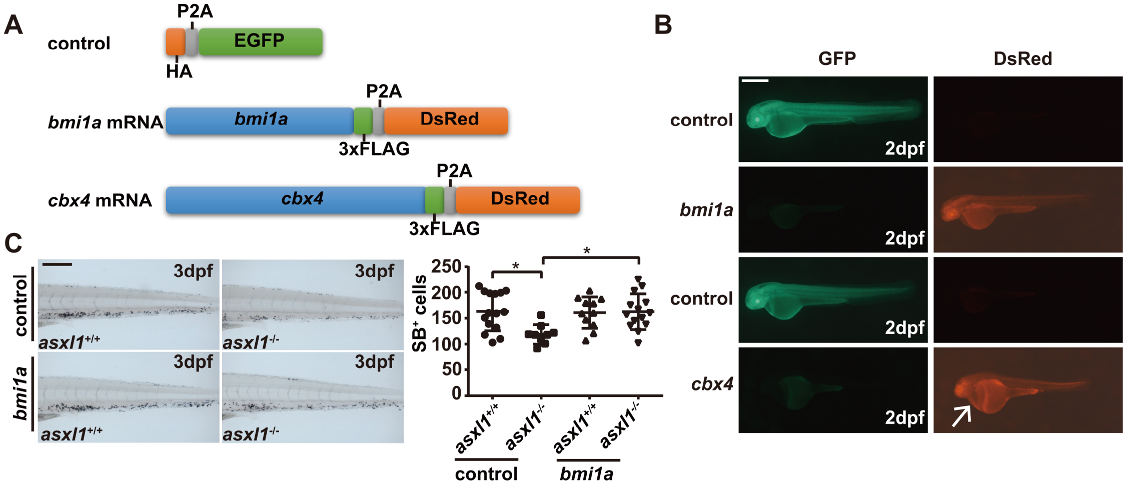

Supplement: Supplementary file 1 — Supplemental materials [file 41375_2021_1121_MOESM1_ESM.pdf]
